# National Stereotypes and Robots' Perception: The “Made in” Effect

**DOI:** 10.3389/frobt.2019.00021

**Published:** 2019-04-09

**Authors:** Nicolas Spatola, Nolwenn Anier, Sandrine Redersdorff, Ludovic Ferrand, Clément Belletier, Alice Normand, Pascal Huguet

**Affiliations:** ^1^CNRS UMR 6024, LAPSCO, Université Clermont Auvergne, Clermont-Ferrand, France; ^2^Laboratoire Psychologie du Développement Cognitif, Université de Fribourg, Fribourg, Switzerland

**Keywords:** social robots, stereotypes, anthropomorphism, humanization, social categorization

## Abstract

In the near future, the human social environment worldwide might be populated by humanoid robots. The way we perceive these new social agents could depend on basic social psychological processes such as social categorization. Recent results indicate that humans can make use of social stereotypes when faced with robots based on their characterization as “male” or “female” and a perception of their group membership. However, the question of the application of nationality-based stereotypes to robots has not yet been studied. Given that humans attribute different levels of warmth and competence (the two universal dimensions of social perception) to individuals based in part on their nationality, we hypothesized that the way robots are perceived differs depending on their country of origin. In this study, participants had to evaluate four robots differing in their anthropomorphic shape. For each participant, these robots were presented as coming from one of four different countries selected for their level of perceived warmth and competence. Each robot was evaluated on their anthropomorphic and human traits. As expected, the country of origin's warmth and competence level biased the perception of robots in terms of the attribution of social and human traits. Our findings also indicated that these effects differed according to the extent to which the robots were anthropomorphically shaped. We discuss these results in relation to the way in which social constructs are applied to robots.

## Introduction

In the next few years, robots will be part of our everyday lives (Fujita and Kitano, 1998; Goldberg, 2001; Lee et al., 2010; Kee, 2011). Technological progress in robotics is accelerating exponentially and the introduction of social robots in some regions of the world is already a reality. Much effort has also been devoted to increasing the acceptance of social robots by giving them more and more human features, such as a national identity and citizenship (e.g., the robot Sophia, which was granted Saudi Arabian citizenship) (De Graaf and Ben Allouch, 2013). However, it remains unclear how attributing human social constructs such as nationality to robots impacts the way they are perceived by people in general. One's country of origin is certainly far from being an anodyne piece of information in the eyes of many individuals. Nationality is almost always associated with positive or negative social stereotypes which may strongly bias our social perception (Fiske et al., 2007). Previous research has shown that under certain circumstances, human social representations (including social stereotypes) are used to evaluate social robots (Siegel et al., 2009) and reduce the conceptual distance between them and us (Eyssel et al., 2011). Thus, attributing citizenship to robots might have some consequences on the way individuals perceive them. The present research examines how humans evaluate robots as a function of their country of origin and their degree of anthropomorphism.

### The Effect of Robots' Nationality

Competition is heating up between major economies that are racing to develop the most cutting-edge technology in robotics. The “made in” label (i.e., the country of manufacture, production, or growth of an object) is therefore an important item of information regarding robots. In connection with human beings, citizenship as well as country of origin, constitutes information that is not psychologically neutral and is frequently used as a basis for social categorization (Fiske et al., 2018). Indeed, to navigate in a complex environment, individuals spontaneously, and effortlessly group social targets into categories based on features such as age, gender, ethnicity (Dunn and Dawson, 1951; Carpenter et al., 2009; O'Connor, 2017) as well as disease and disability (Stangor and Lange, 1994; Kao, 2000; van Knippenberg and Dijksterhuis, 2000). Individuals hold stereotypes concerning many social categories, i.e., socially shared beliefs about the members of a given social group or category, most notably regarding their personality traits, intelligence level, skills and competences, preferences, intentions, typical behaviors, etc. (Stangor and Lange, 1994). Categorizing a social target almost always activates the related stereotypes that guide individuals' attitudes and behaviors toward outgroup members (van Knippenberg and Dijksterhuis, 2000) as well as toward the self via a self-categorization process when the stereotypes describing the groups or categories to which the individuals belong are made salient (Schmader, 2010). National stereotypes refer to beliefs about distinctive traits (such as those mentioned earlier) that members of the same country are thought to share (Macrae et al., 1996; Madon et al., 2001) when compared with geographically close or competing countries (Kao, 2000; Terracciano et al., 2005; Durik et al., 2006; Schmader, 2010; Brescoll, 2016). Stereotypes at this scale are especially powerful and do indeed contribute greatly to fueling major intergroup conflicts, prejudice, and discrimination (Tajfel, 1982).

To our knowledge, very few studies have examined the consequences of the concept of nationality applied to robots (Lee et al., 2005). Eyssel and Hegel (2012) showed that people may rate robots that share their own nationality more favorably, thus demonstrating the existence of an in-group favoritism phenomenon similar to that typically found in human-human relationships (Tajfel, 1982; Buttelmann and Böhm, 2014; Häring et al., 2014; Rutland et al., 2015). However, we suspect that the influence of robots' nationality is not limited to in-group favoritism, but may also play a major role in the way robots are perceived based on national stereotypes. Indeed, research into human intergroup relations has repeatedly found not only that individuals favor their in-group but that they also hold negative beliefs (i.e., stereotypes) regarding ethnic, national, and social outgroup members (Madon et al., 2001). Such negative beliefs thus contribute to the existence of prejudice and discrimination. The present paper addresses this issue by investigating how information about the different robots' country of manufacture affects the way people perceive them.

### Anthropomorphism and Robot Social Categorization

The anthropocentric projection of human attributes onto non-human targets, for example in the form of stereotypical traits based on nationality as well as emotions, logical thoughts, and consciousness has been described at the theoretical level as anthropomorphism (Epley et al., 2007). The tendency to anthropomorphize humanoid robots does not appear to depend, mainly, on the frequency of human-robot interactions but is thought to be spontaneous: most people automatically appear to attribute human logic to robots (Häring et al., 2014). Given this powerful anthropomorphic tendency, one may wonder whether people's social beliefs can filter their perception of humanoid robots (Nass et al., 1996).

Previous research has shown that social characteristics that are generally attributed to human beings can be projected onto robots, thereby orienting expectations about their abilities and roles (Eagly and Steffen, 1984). In other words, individuals would tend to categorize robots (as they also do humans) on the basis of social characteristics (i.e., social categorization), and to apply to robots the attributes associated with these categories (i.e., stereotypes). For example, Tay et al. (2014) showed that a robot with a male name was preferred for security roles, whereas the same robot with a female name was preferred for healthcare roles (in line with stereotypes associating certain jobs with a particular gender). According to some authors, imbuing robots with anthropocentric social characteristics (e.g., gender) in this way would (1) make the contact more fluid and the interaction more intuitive (Powers and Kiesler, 2006; Eyssel and Hegel, 2012), and (2) also illustrate a will to reduce the distance between robots and human by using basic social characteristics (Carpenter et al., 2009). The anthropomorphic attributions and perceived conceptual distance between us and robots could actually be one of the major determinants of the acceptance of robots by facilitating their social categorization (Stangor and Lange, 1994), but may also favor the emergence of prejudices and behaviors that are prohibited in dealings with other humans (Nomura et al., 2006; Chopra and White, 2011; Spatola and Urbanska, 2018). However, the question of whether a robot's country of manufacture may also lead people to perceive it in the light of the national stereotypes associated with the country remains unknown. One's nationality is not a neutral piece of information for human social cognition and may actually be used to justify inequality and injustice, and sometimes leads to discriminatory behaviors targeting a specific part of the population (Bonilla-Silva, 2006), even if unconsciously (Amodio, 2014). Assuming that such information can be assimilated to a form of nationality, then robots' country of manufacture may activate related national stereotypes whose content and valence may influence the way humans perceive them.

According to Fiske et al. (2007) and Dupree and Fiske (2017) most of the existing stereotypes associated with social categories (including nationality) are distributed along two main dimensions: warmth (e.g., sincerity, trustworthiness, morality) and competence (e.g., ambition, confidence). The warmth dimension predicts active behaviors such as helping (high warmth) or attacking (low warmth). The competence dimension predicts passive behaviors such as association (high competence) or neglect (low competence). The valence (positive vs. negative) and content (e.g., psychological traits and behaviors) of social stereotypes therefore depend greatly on the degree of perceived warmth and competence associated with the groups involved. Members of social groups stereotyped as warm and competent are perceived much more positively than members of social groups stereotyped as cold and incompetent. As national affiliations are considered to constitute social groups, knowing a robot's country of manufacture and holding stereotypes about this country may therefore induce a stereotypical view of the robot by moderating the degree of perceived warmth and competence.

### Experiment and Hypotheses

The present study investigates whether information about robots' country of manufacture and the degree of physical anthropomorphism (i.e., their resemblance to humans in terms of shape) influence the way they are perceived in terms of warmth and competence as well as their humanization. The participants had to evaluate four different robots. Depending on the experimental condition, these four robots were presented as developed in one of four different countries that we selected on the basis of their differences along the two main dimensions of stereotypes (i.e., warmth and competence; Fiske et al., 2007). A pretest was conducted in order to select four countries whose positions along the two dimensions of warmth and competence were the most contrasted (see Supplementary Presentation 1). We hypothesized that (1) the participants' perception of robots should conform to the stereotype generally associated with their country of origin. For instance, robots made in stereotypically warm and competent countries should be seen as warmer than an equally competent as robots made in stereotypically cold and competent countries. This stereotypical view of robots, we reasoned, should depend on (2) their anthropomorphization and should (3) moderate the perceived Human-Robot conceptual distance.

## Methods

### Procedure

**Figure 1:**
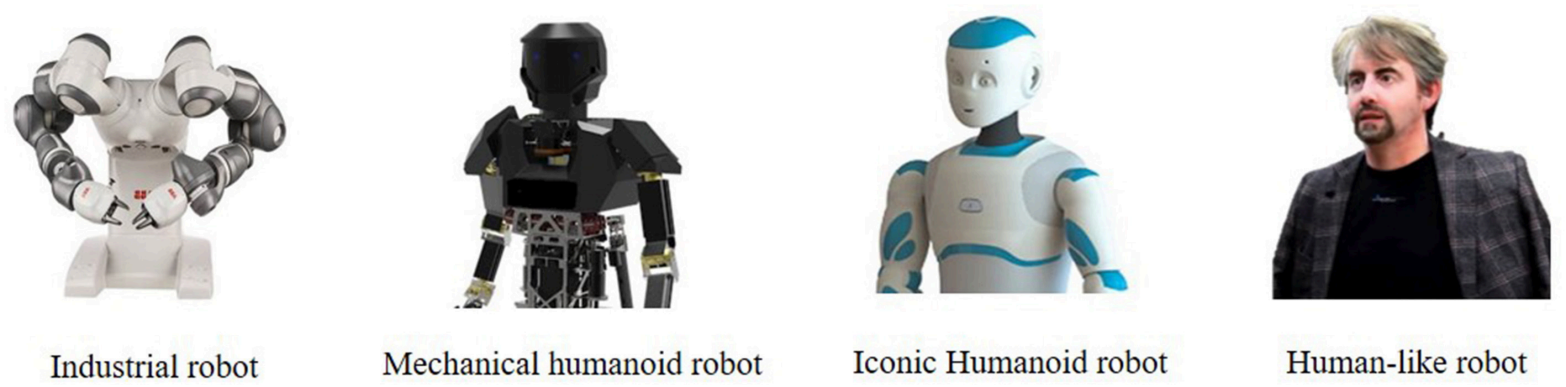
Pictures of four robots presented to the participants from the less (left) to the most (right) strong human-like appearance.

Qualtrics Survey online software was used to design and distribute the questionnaire. The participants (113 women and 26 men, all French^1^) were recruited online via a university mailing list and participated on a voluntary basis (*M*_age_ = 21.7, *SD* = 5.0). They were informed that they would have to evaluate the design of four different robots (i.e., industrial robot, mechanical humanoid robot, iconic humanoid robot, human-like robot, see Figure 1) with the aim of improving their design.

These robots were actually representative of different levels of anthropomorphism from the Duffy taxonomy (Duffy, 2003). The country of manufacture was manipulated at the between-participants level. Each participant thus saw, in a random order, the same images of four different robots, which were described as having been assembled in the country corresponding to the condition to which the participant was assigned (Canada, Spain, Russia, Turkey). According to Papadopoulos (1993) and others (for a review, see Al-Sulaiti and Baker, 1998), the country of assembly does indeed correspond to the country of perceived origin. Among these countries, according to our pretest (see Supplementary Presentation 1), Canada was representative of the high warmth/high competence combination, Spain was representative of high warmth/low competence, Russia was representative of low warmth/high competence and Turkey was representative of low warmth/low competence.

### Measures

#### Country Priming and Control Measures

To reinforce the activation of the representation of the warmth and competence of the country, the participants had to respond to two questions: “How much do you know about the technological level of [the country]?” and “How much do you know about the technological level of robotics in [the country]?” (the scores ranged from 0: no knowledge, to 100: perfect knowledge). Then the participants had to evaluate the correspondence between the country assigned to them at the beginning of the experiment and different stereotypical traits characterizing the warmth and competence dimensions (Kervyn et al., 2008).

#### The Robotic Social Attributes Scale (RoSAS) (Carpinella et al., 2017)

This scale makes it possible to evaluate robots on the dimensions of warmth (e.g., “emotional”), competence (e.g., “interactive”), and discomfort (i.e., “I find this robot scary”). This scale has been standardized to measure the social perception of robots based on their appearance. For each dimension, the participants had to indicate whether they thought the different characteristics fitted the presented robot (from 1 “does not fit at all” to 9 “totally fits,” Cronbach's α = 0.77).

#### Conceptual Human-Robot Distance

The participants also completed the humanness scale based on Haslam's dehumanization taxonomy (Haslam, 2006). The Humanization-Dehumanization process consists in perceiving or treating people as more or less human. This process involves two bi-dimensional constructs: (1) animalistic dehumanization as opposed to human uniqueness (e.g., Amorality/Moral sensibility), which distinguishes humans from other animals; (2) mechanistic dehumanization as opposed to human nature (e.g., Rigidity/Cognitive openness), which represents typical characteristics central to human beings and the distance between machines and humans. The participants rated each robot from 1 to 6 on each bi-dimensional construct (e.g., 1 “Amorality”/6 “Moral sensibility,” Cronbach's α = 0.89).

#### Demographic Information

Finally, the participants were invited to make comments about the study and to provide information about their age, gender, and country of origin. This information was collected at the end of the study, so as not to prime the participant's own country of origin before data collection.

## Results

See Supplementary Table 1.

### Country Priming Manipulation Check

We first checked the participants' perceptions of the tested countries in terms of warmth/competence. As expected, Spain and Canada were seen as warmer than Russia and Turkey [*F*_(1, 170)_ = 78.66, *p* < 0.001, $\eta_{p}^{2}$ = 0.32]. However, and contrary to what we observed during the pre-test, Canada and Russia were not seen as more competent than Spain and Turkey [*F*_(1, 170)_ = 3.34, *p* = 0.069, $\eta_{p}^{2}$ = 0.02]. No link was found between the countries' perceived warmth and competence and their level of general technology and robotic technology (all *p*_*s*_ > 0.05). The results showed that participants' knowledge was lower than the 25% threshold for both technological [*M* = 16.6, *SD* = 22.0, *t*_(170)_ = −4.98, *p* < 0.001] and robotic technology [*M* = 5.47, *SD* = 12.1, *t*_(170)_ = −19.53, *p* < 0.001], indicating that they were probably not well informed about this topic.

### Main Analyses

We started by confirming the factorial structure of RoSAS and the Humanization-Dehumanization scale using factorial analyses. For the RoSAS scale, factor loading was 0.803, with the 3 dimensions Warmth, Competence, and Discomfort explaining 69% of the variance. For the Humanization scale, factor loading was 0.905, and the 2 dimensions Human Nature and Human Uniqueness explained 67% of the variance. We then conducted a repeated-measures analysis including each dependent variable of robot social attributes and humanization (the three dimensions of the RoSAS scale and the two dimensions of the Humanization–Dehumanization scale) and the type of robots (four levels of anthropomorphism) as within factors, while the level of warmth (high/low) and competence (high/low) of the countries were included as between factors.

Because of the similarity in the naming of the social dimensions of warmth and competence related to the countries and the same dimensions on the RoSAS scale we will refer to the latter as warmth^RoSAS^, competence^RoSAS^, and discomfort^RoSAS^.

#### The Robotic Social Attributes Scale

**Figure 2:**
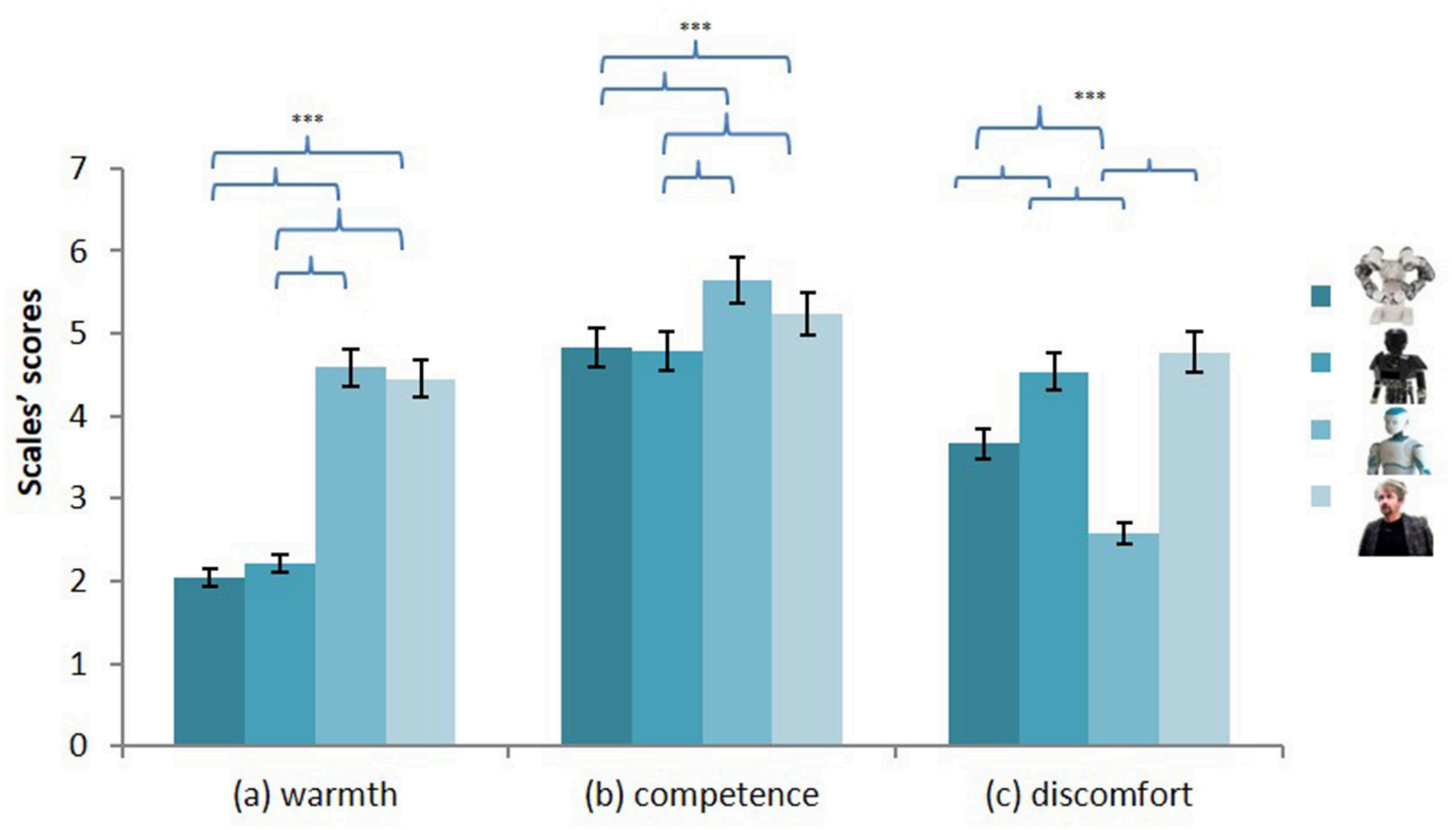
Level of warmth (a) competence (b) and discomfort (c) attributed to the four robots. ***p < 0.001.

We conducted a multivariate analysis including the type of robots as IV (industrial, mechanical, iconic, human-like) and the RoSAS dimensions as DVs (warmth^RoSAS^, competence^RoSAS^, discomfort^RoSAS^). The type of robot had a significant effect on perceived warmth [*F*_(3, 137)_ = 152.26, *p* < 0.001, η^2^ = 0.53], competence [*F*_(3, 137)_ = 19.95, *p* < 0.001, $\eta_{p}^{2}$ = 0.13] and discomfort [*F*_(3, 137)_ = 61.90, *p* < 0.001, $\eta_{p}^{2}$ = 0.31, see Figure 2].

##### Warmth^*RoSAS*^

With regard to the perceived warmth^RoSAS^ of the robots, no difference was found between the industrial (1) and the mechanical robots (2) [*t*_(138)_ = −1.29, *p* = 0.200, *d*_*RM*_ = 0.11]. There was also no difference between the iconic (3) and the human-like (4) robots [*t*_(137)_ = 1.04, *p* = 0.301, *d*_*RM*_ = −0.09]. However, the industrial robot and the mechanical robot were seen as less warm^RoSAS^ than the iconic [*t*_1.3__(137)_ = −16.28, *p* < 0.001, *d*_*RM*_ = 1.41; *t*_2.3__(137)_ = −14.51, *p* < 0.001, *d*_*RM*_ = 1.26] and the human-like robot [*t*_1.4__(138)_ = −13.46, *p* < 0.001, *d*_*RM*_ = 1.16; *t*_2.4__(138)_ = −13.47, *p* < 0.001, *d*_*RM*_ = 1.17].

##### Competence^RoSAS^

While there was no difference between the industrial (1) and the mechanical robot (2) [*t*_(138)_ = 0.50, *p* = 0.615, *d*_*RM*_ = −0.04], these two robots were judged as less competent^RoSAS^ than the iconic (3) [*t*_1.3__(137)_ = −6.86, *p* < 0.001, *d*_*RM*_ = 0.59; *t*_2.3__(137)_ = −7.24, *p* < 0.001, *d*_*RM*_ = 0.62] and human-like (4) robots [*t*_1.4__(138)_ = −2.73, *p* = 0.007, *d*_*RM*_ = 0.23; *t*_2.4__(138)_ = −3.32, *p* = 0.001, *d*_*RM*_ = 0.26]. The iconic robot was also seen as more competent^RoSAS^ than the human-like robot [*t*_(137)_ = 3.33, *p* = 0.001, *d*_*RM*_ = −0.28].

##### Discomfort^RoSAS^

Regarding the discomfort^RoSAS^ score, there was no difference between the mechanical (2) and the human-like (4) robots [*t*_(138)_ = −1.25, *p* = 0.215, *d*_*RM*_ = 0.10]. However, the mechanical and the human-like robots were seen as more discomfort-provoking than the industrial (1) [*t*_2.1__(138)_ = 4.99, *p* < 0.001, *d*_*RM*_ = −0.43; *t*_4.1__(138)_ = 6.07, *p* < 0.001, *d*_*RM*_ = −0.51] and iconic (3) [*t*_2.3__(137)_ = 11.66, *p* < 0.001, *d*_*RM*_ = −1.02; *t*_4.3__(138)_ = 12.22, *p* < 0.001, *d*_*RM*_ = −1.05] robots. The participants also attributed less discomfort^RoSAS^ traits to the iconic robot than to the industrial one [*t*_(138)_ = 6.07, *p* < 0.001, *d*_*RM*_ = −0.56].

##### Country of origin

**Figure 3:**
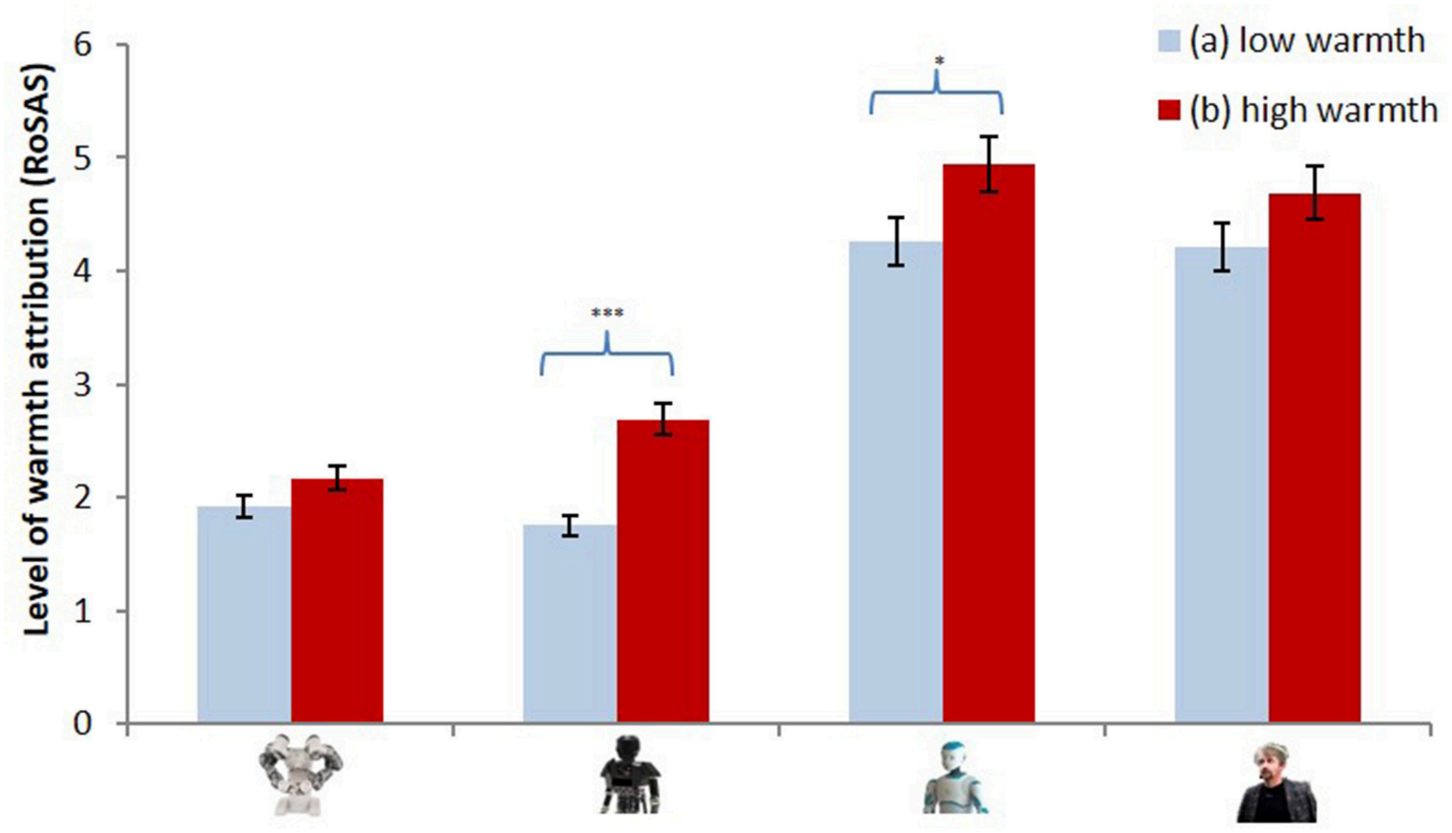
Level of warmth traits attributed to the four robots in a low warmth (a) vs. high warmth (b) country. *p < 0.05, ***p < 0.001.

On the warmth dimension, regression analysis showed a positive relation between the countries' perceived warmth and the robots' perceived warmth^RoSAS^, *b* = 0.19, *t*_(138)_ = 2.21, *p* = 0.029, $\eta_{p}^{2}$ = 0.04. Thus, the warmer a country was perceived to be, the warmer the perception of the corresponding robot. Regarding the evaluation of the warmth of the countries, we conducted a repeated-measures analysis including the four warmth^RoSAS^ scores for the robots as within factors and the distinguishing variable for high-warmth countries (Canada and Spain) vs. low-warmth countries (Russia and Turkey) as between factor. We found a significant warmth^RoSAS^ x countries' warmth level interaction, *F*_(3, 134)_ = 4.75, *p* = 0.041, $\eta_{p}^{2}$ = 0.06. A decomposition of this level-of-warmth interaction showed that the mechanical humanoid robot was seen as warmer^RoSAS^ (e.g., more emotional) when presented as made in Spain or Canada (i.e., high-warmth countries), *F*_(1, 136)_ = 20.04, *p* < 0.001, $\eta_{p}^{2}$ = 0.13 (see Figure 3). This effect was also significant for the iconic humanoid robot, *F*_(1, 136)_ = 4.52, *p* = 0.035, $\eta_{p}^{2}$ = 0.03.

We conducted a similar regression analysis with the countries' perceived competence and the robots' perceived competence^RoSAS^. Overall, the more competent the country was perceived to be, the greater the competent^RoSAS^ rating of the corresponding robot was, *b* = 0.17, *t*_(138)_ = 2.06, *p* = 0.042, $\eta_{p}^{2}$ = 0.03. However, when we look at each robot separately, the high vs. low competence rating of the country of origin no longer affected the perception of each robot's competence (all *p*_*s*_ > 0.05).

#### The De-humanization Scale

**Figure 4:**
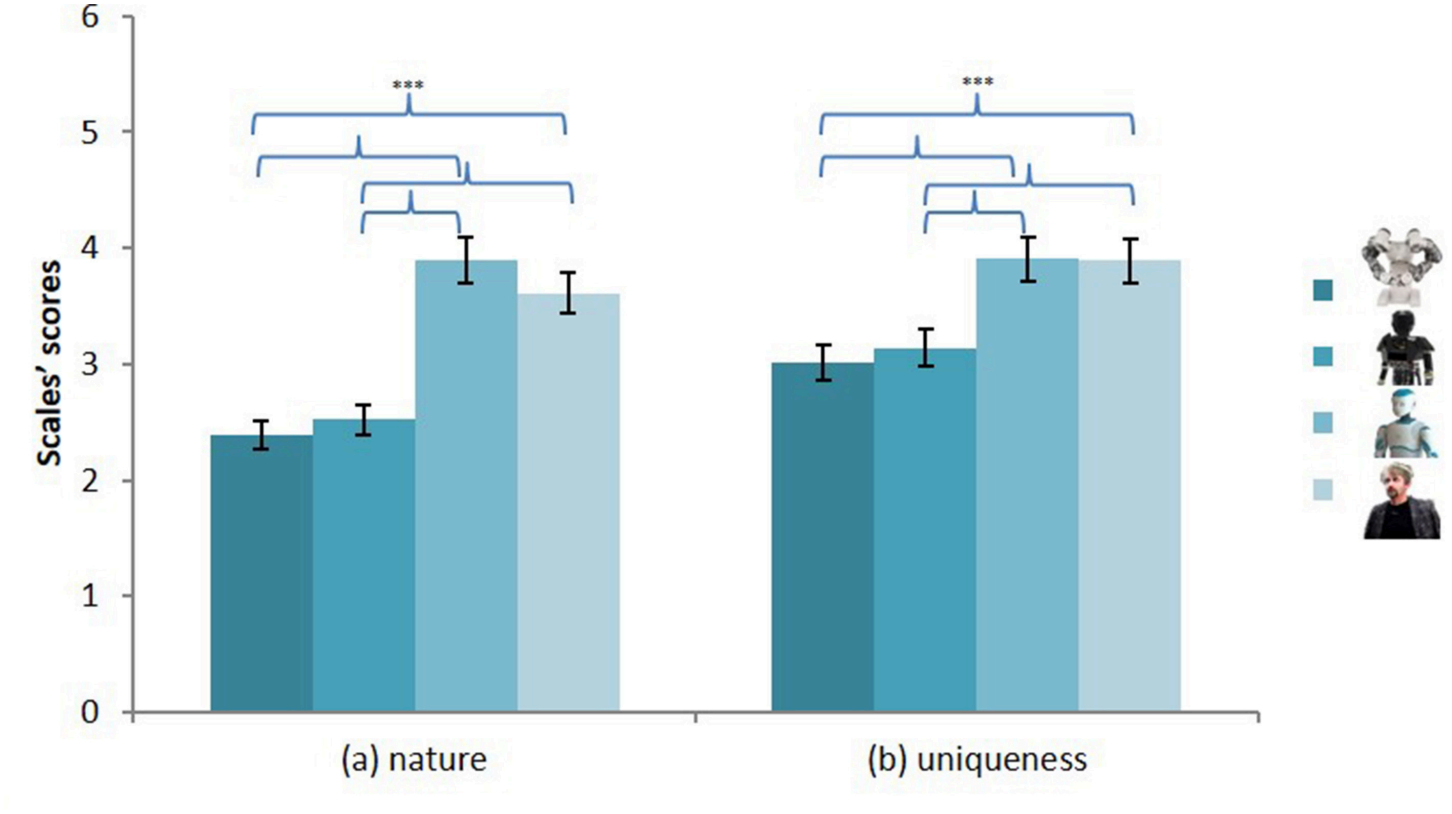
Level of human nature (a) and human uniqueness (b) traits attributed to the four robots. ***p < 0.001.

To investigate the dehumanization scale we conducted a repeated-measures analysis including the type of robot (industrial, mechanical, iconic, human-like) and the dimensions of the de-humanization scale (human nature, human uniqueness). The type of robot had a significant effect on the rating of the level of human nature, opposing the attribution of mechanical traits (e.g., passive) to human nature (e.g., emotional) [*F*_(3, 137)_ = 71.71, *p* < 0.001, $\eta_{p}^{2}$ = 0.34], and on that of the level of human uniqueness, opposing the attribution of animal traits (e.g., irrational) to uniquely human traits (e.g., moral) [*F*_(3, 137)_ = 27.29, *p* < 0.001, $\eta_{p}^{2}$ = 0.17] (Figure 4).

##### Human nature

With regard to the attribution of human nature traits, there was no difference between the industrial (1) and the mechanical robots (2) [*t*_(138)_ = 0.20, *p* = 0.839, *d*_*RM*_ = −0.01]. Also, the participants attributed more human nature traits to the iconic (3) [*t*_1.3__(137)_ = −10.93, *p* < 0.001, *d*_*RM*_ = 0.93; *t*_2.3__(137)_ = −11.31, *p* < 0.001, *d*_*RM*_ = 1.07] and the human-like (4) robot [*t*_1.4__(138)_ = −8.10, *p* < 0.001, *d*_*RM*_ = 0.69; *t*_2.4_(138) = −9.66, *p* < 0.001, *d*_*RM*_ = 0.82] than they did to the industrial and mechanical robots. The iconic robot was also seen as more human than the human-like robot [*t*_(137)_ = 2.54, *p* = 0.012, *d*_*RM*_ = −0.21].

##### Human uniqueness

Concerning the uniquely human traits, no difference was found between the industrial (1) and the mechanical robots (2) [*t*_(138)_ = 0.91, *p* = 0.367, *d*_*RM*_ = −0.08]. There was also no difference between the iconic (3) and the human-like (4) robots [*t*_(137)_ = 0.16, *p* = 0.870, *d*_*RM*_ = −0.01]. However, the industrial robot and the mechanical robot were seen as less human than the iconic [*t*_1.3__(137)_ = −5.41, *p* < 0.001, *d*_*RM*_ = 0.47; *t*_2.3__(137)_ = −7.04, *p* < 0.001, *d*_*RM*_ = 0.60] and the human-like robot [*t*_1.4__(138)_ = −5.37, *p* < 0.001, *d*_*RM*_ = 0.46; *t*_2.4__(138)_ = −7.48, *p* < 0.001, *d*_*RM*_ = 0.64].

We compared each robot's human nature and uniqueness evaluation with the central point of the bi-dimensional scale (−3 to −1 for dehumanization, 1 to 3 for humanization). The results showed that all the robots except for the iconic humanoid one [*t*_*nature*__(137)_ = −1.04, *p* = 0.299; *t*_*uniqueness*__(138)_ = 0.24, *p* = 0.239] were assigned to the dehumanization side of the scale (all *p*_*s*_ < 0.05). The evaluation of the iconic humanoid robot did not differ from the central value of the scale.

##### Country of origin

We ran a repeated-measures analysis with the distinguishing variable for high-warmth countries (Canada and Spain) vs. low-warmth countries (Russia and Turkey) and competence (high vs. low) as between factors and the type of robot as a within factor.

Regarding the attribution of human nature traits, the results showed a main effect of the countries' warmth, *F*_(1, 136)_ = 4.29, *p* = 0.040, $\eta_{p}^{2}$ = 0.03. The participants attributed more human nature traits to robots presented as made in high-warmth countries (Canada or Spain) than in low-warmth countries (Russia or Turkey). This main effect was further qualified by an interaction with the type of robot, *F*_(3, 133)_ = 2.70, *p* = 0.045, $\eta_{p}^{2}$ = 0.02. A decomposition of this interaction demonstrates that the mechanical robot was judged as having more human nature traits when thought to be made in high-warmth countries, *F*_(1, 136)_ = 18.38, *p* < 0.001, $\eta_{p}^{2}$ = 0.20. No other effect reached significance.

**Figure 5:**
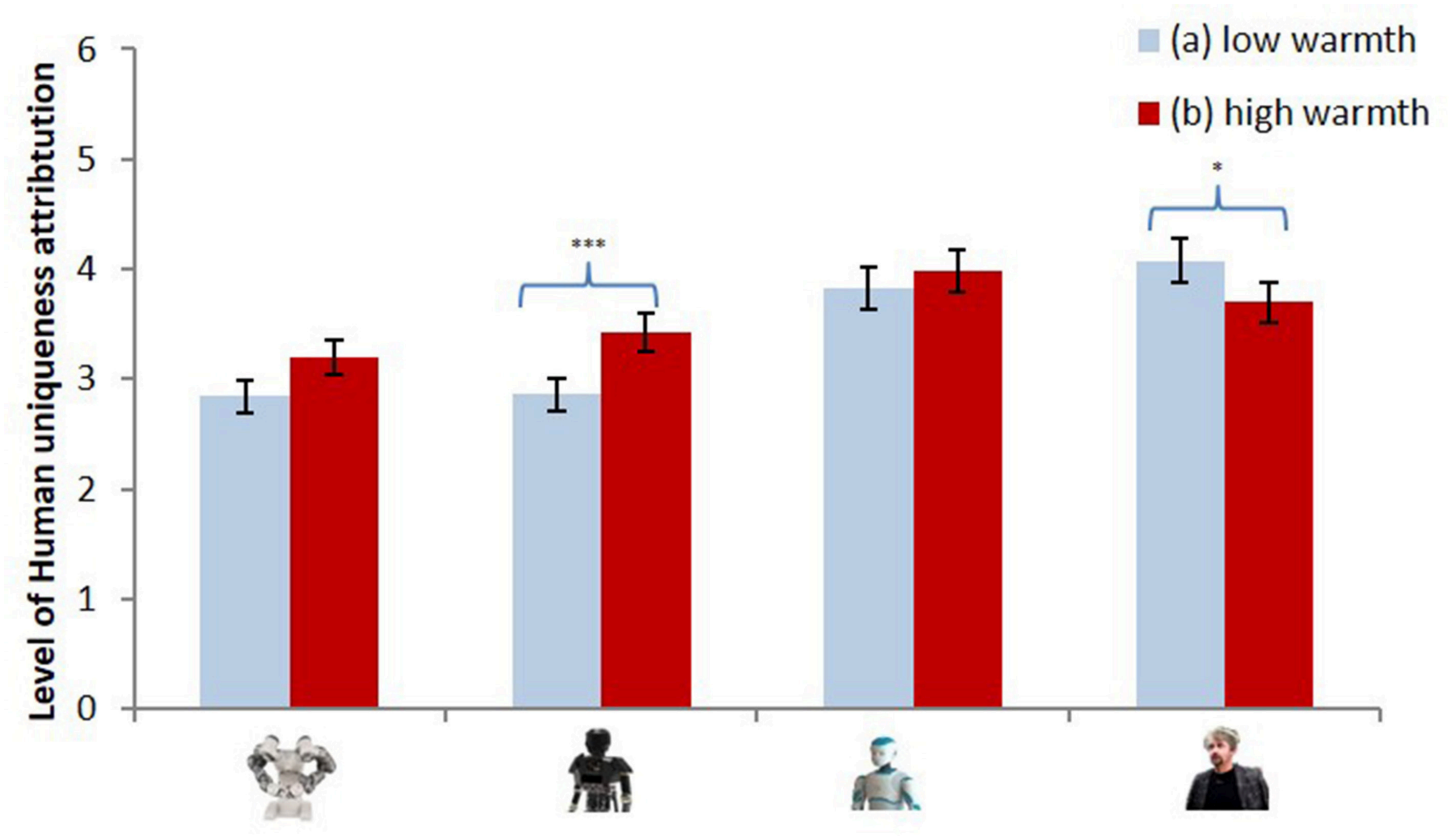
Level of human uniqueness attributed to the four robots presented as developed in a low warmth (a) vs. high warmth (b) country.

Regarding the attribution of human uniqueness traits, the results showed a significant interaction between countries' warmth and the type of robot, *F*_(3, 133)_ = 4.12, *p* = 0.008, $\eta_{p}^{2}$ = 0.09; cf. Figure 5. Decomposition analyses showed that the participants attributed more uniquely human traits to the mechanical robot (2) [*F*_(1, 135)_ = 9.62, *p* = 0.001, $\eta_{p}^{2}$ = 0.08] and, interestingly, less to the human-like robot (4) [*F*_(1, 135)_ = 7.00, *p* = 0.009, $\eta_{p}^{2}$ = 0.05] when they were presented as coming from high-warmth (i.e., Spain, Canada) rather than low-warmth countries (i.e., Turkey, Russia). The results also pointed to a significant interaction between countries' competence and the type of robot, *F*_(3, 133)_ = 2.17, *p* = 0.048, $\eta_{p}^{2}$ = 0.06. The mechanical humanoid robot (2) was seen as more uniquely human when presented as coming from a high-competence rather than a low-competence country, *F*_(1, 135)_ = 12.37, *p* = 0.001, $\eta_{p}^{2}$ = 0.08.

## Discussion

We hypothesized that (1) the perception of a robot should conform to the stereotype generally associated with its country of origin and that this effect (2) should be strengthened by the degree of anthropomorphization, and (3) would also impact the perceived Human-Robot conceptual distance.

### Social Categorization

Our main hypothesis was that robots' country of origin influences the way individuals perceive them. We argued that, through social categorization, individuals should attribute to robots the stereotypical traits associated with their country of origin. We focused on two main dimensions grouping most of the existing stereotypes associated with social categories: warmth and competence (Fiske et al., 2007; Dupree and Fiske, 2017).

First, and independently of the effect of the country of origin, the results showed that the four robots presented to the participants, which differed in terms of anthropomorphism, were not perceived as equally warm and competent. The two less anthropomorphized robots (the industrial and the mechanical robots) were perceived as less warm than the other two (the iconic and the human-like robots). They were also perceived as less competent, indicating that anthropomorphic shape interacts with the perceived technological level of robots. Thus, the more anthropomorphic a robot is, the more technologically advanced, and consequently more competent, it is perceived to be (Duffy, 2003). It is worth noting that there was a slight difference on the competence attribution between the iconic and the human-like robot. The iconic robot was actually perceived as being even more competent than the human-like robot. Competence is not the only dimension on which the participants rated the iconic robot more highly than the human-like robot. Despite its higher level of anthropomorphism, the human-like robot was also attributed less human nature traits than the iconic robot. Altogether, these first observations suggest that the level of anthropomorphism does influence the projection of human characteristics onto robots but that the importance of this factor becomes less at very high levels of anthropomorphism. Such results are consistent with the uncanny valley theory (Mori et al., 2012). This theory proposes that the more similar an android robot is to a human being, the more it will be accepted. However, this is only true up to a certain point at which its small imperfections become too disturbing, leading to a fall-off in acceptance. In support of this rationale, the participants in the present study reported high levels of discomfort not only in connection with the mechanical robot but also the human-like robot.

In addition to these prior differences between robots of varying degrees of anthropomorphism, the country of origin also modified the participants' perception of the robots' warmth, competence, human nature and human uniqueness. Robots which were presented as assembled in high-competence countries were attributed greater competence, independently of their level of anthropomorphism. However, only the mechanical and the iconic robots were attributed greater warmth when presented as assembled in high-warmth countries. Regarding the attribution of human nature and uniqueness, only the mechanical robot was attributed more of these traits when presented as originating from high-warmth or high-competence countries. In sum, except for the attribution of competence, the country-of-origin effect largely depends on the robots' level of anthropomorphism. On the one hand, the least anthropomorphized robot–the industrial robot–was never perceived differently as a function of its country of origin. Anthropomorphism is a well-known antecedent of perception in human-robot interactions (Fujita and Kitano, 1998; Goldberg, 2001; Powers and Kiesler, 2006; Carpenter et al., 2009; Lee et al., 2010; Kee, 2011; De Graaf and Ben Allouch, 2013). Anthropomorphic shape facilitates the attribution of human characteristics to robots (Ferrari et al., 2016) and the same seems to be the case for the attribution of social constructs. It is plausible that the projection of social constructs, such as national stereotypes, onto robots requires a minimal level of anthropomorphism that the industrial robot, which is headless, does not reach. On the other hand, the most anthropomorphized robot–the human-like robot–was not perceived differently as a function of its country of origin, except in the case of one unexpected result: the human-like robot was attributed *less* human uniqueness when presented as being assembled in high-warmth countries. As stated above, the human-like robot may actually fall into the uncanny valley, thereby creating psychological discomfort. According to this view, when facing such a disturbing robot, individuals may rather focus on the perceptual dissimilarities between it and humans. Such a “highly critical” mindset could prevent the projection of human social constructs onto the human-like robot. In sum, the present results suggest that more than being an in-group/out-group process (Madon et al., 2001; Eyssel and Kuchenbrandt, 2012), the social categorization of robots seems to be based on sharing a group membership but, more generally, on the salience of any characteristics that make it possible to project human social knowledge. Here, the attribution seems to be based on (1) the cultural representation of the foreign country of assembly and (2) the attribution of human traits according to the level to which the robots are anthropomorphized. Further research will address this possibility. We also found that the more competent the country of manufacture was perceived as being, the greater the level of competence attributed to the robots themselves. However, we did not find any modulation specific to the countries or to the type of robot. This lack of results could be explained by the lack of any significant difference in the competence rating between high and low-competence countries (in contrast with the pre-test results).

### A Country-of-Origin Effect?

It could be argued that the present results are entirely consistent with the country-of-origin effect (COE) previously described in the literature (Cai, 1994; Lusk et al., 2006). Previous research has defined the COE as a psychological effect describing how consumers' attitudes, perceptions and purchasing decisions are influenced by products' country-of-origin labeling (Rezvani et al., 2012). COE appears to be one of the main determinants of the perception and attribution of positive or negative valence regarding the quality or reliability of objects (Peterson and Jolibert, 1995; Verlegh and Steenkamp, 1999). According to this view, previous results on social attribution in the in/out-group paradigm (Macrae et al., 1996; Madon et al., 2001; Lee et al., 2005; Terracciano et al., 2005) could be reduced to consumer ethnocentrism. Such a view of the COE does not strictly apply to the present results. First, robots are not considered as simple technological objects (Duffy, 2003; Epley et al., 2007). Second, this proposal is inconsistent with our results. The robots' countries of origin were all different from those of the participants. As a result, no ethnocentric bias could have emerged (i.e., positive in-group bias), as evidenced by previous studies (Eyssel et al., 2011). Second, for a strict COE to emerge in our experiment, the participants would have had to demonstrate considerable knowledge about the level of technology and robotics in the various countries, since this is a prerequisite for technological discrimination (Kotler and Gertner, 2002). This was not the case. Third, the “COE” is due to technical characteristics such as safety, and robustness, but not to social characteristics such as warmth. Fourth, in robot studies, this proposal underestimates the specificity of the attribution of human characteristics to robots. Despite this, the more anthropomorphic robots are, the more humans attribute human social constructs such as culture or open-mindedness (items present in the Haslam taxonomy) (Haslam, 2006) to them.

### Difference Between Anthropomorphism and De-humanization Scale

The present results show that the perceived warmth and competence of a country of origin moderates the attribution of human traits as measured by the dehumanization scale (Haslam, 2006; Haslam and Loughnan, 2014) and the *robotic social attributes scale* (RoSAS). The unexpected effect found for the human-like robot is interesting in that it helps us to understand the perception of humanoid robots (e.g., geminoid). While there was no impact on the attribution of anthropomorphism traits, the human-like robot was considered as less human when made in a high-warmth country than when it was made in a low-warmth country. In line with the uncanny valley theory, the human-like robot seems to have elicited psychological discomfort. When told that the human-like robot was assembled in a high-warmth country, the participants might have expected such “sympathetic citizens” to be more adept in designing a “pleasant robot.” However, the uncomfortable experience of the uncanny valley may have violated the expectations of the participants, who might then have reacted by refusing to attribute human traits to the human-like robot.

These results could illustrate two associated processes in the concept of anthropomorphism: first, perceived anthropomorphism, as the attribution of human characteristics to other non-human entities; and second, humanization, which refers to the phenomenon of distance modulation between robots and humans as a function of social representations and expectations. This concept of humanization is supported by several results from the literature on HRI. First, HRI triggers similar behaviors in humans as those observed in human interactions (Riether et al., 2012; Spatola et al., 2018). These similarities suggest common cognitive processes that rely on common brain areas (Wiese et al., 2017). Second, a recent study by Spatola et al. (2018) showed that the positive or negative valence associated with a short interaction with a very simple robot influenced participants' humanization inferences, while robotic social attributes (i.e., anthropomorphic inferences) remained the same. Moreover, after the negative interaction, the mere presence of the robot was sufficient to induce an effect similar to that observed in human-human interactions (Huguet et al., 1999; Augustinova and Ferrand, 2012). The participants' attentional processes were impacted in a subsequent cognitive task performed in a condition in which the robot was merely present, which did not occur in the positive interaction condition. These results echoed previous human-human studies that suggest that individuals perform differently when in the presence of conspecifics (see also Spatola et al., 2019). Taken together, the results suggest that humans could tend to perceive robots as viable social agents and consider their presence as similar to that of humans.

In addition, several fMRI HRI studies argue that the perception of robots is based on systems dedicated to human behavior perception that create a direct link with the motor resonance system. Motor resonance is a perceptual phenomenon (i.e., bottom-up). In the same way that we cannot help but recognize a human face when we perceive it, we cannot help but represent, in motor terms, the actions we perceive (or imagine). For human beings, the information that leads to this activity is fully automatic and therefore not subject to modulation by context. By contrast, for robots, the resonance would naturally be less strong and would be modulated, first, by the perceptual proximity with the human and, second, by contextual information (Chaminade et al., 2005, 2010). This contextual information may include applicable social constructs. Thus, anthropomorphism could rely on and impact the bottom-up process, while humanization would rely on and impact top-down processes, such as the integration of social and contextual information (Waytz et al., 2010) or mentalization (i.e., understanding the mental state that underlies another person's behavior) (Decety et al., 2004; Chaminade et al., 2012). Nonetheless, the concept of humanization as being correlated with anthropomorphism remains unexplored and its relation with anthropomorphism has to be clarified.

Finally, in the desire to promote a more positive HRI in the future, it is important to consider that robots will not be perceived as equivalent on the “humanness” scale (Haslam, 2006) in the light of the associated human social constructs (e.g., national stereotypes). In the human social environment, dehumanization can cause a person's membership of the group of human beings to be underestimated, with humans even being considered as machines (Haslam and Loughnan, 2014). To perceive a person as less human can lead to physical abuse, psychological violence and even slavery (Haslam, 2006; Haslam and Loughnan, 2014; Kteily et al., 2015). This theory illustrates how we can perceive and act with other humans that we consider to be robot-like (Haslam, 2006) and therefore sheds light on the nature of potential HRIs. It is therefore possible that ethical problems may arise with regard to robotics in the future. It is unlikely that we could interact with and consider artificial agents in a more positive way than dehumanized humans without thinking very carefully about the determinants (such as the country of origin) that can favor or inhibit the humanization process. For example, robots from perceived low-warmth countries could be considered as acceptable targets for socially unacceptable behaviors because they would be perceived as more distant from humans than those from high-warmth countries. Could such behaviors reinforce the stereotypical representations associated with the citizens of these countries or certain categories of the population?

### Limitations and Future Research

The use of existing robots could somehow bias participants' responses due to their knowledge about these robots. Even if the participants were all lacking in knowledge of robotic technology, as shown by our control measure, we cannot be sure that they did not know about these existing robot exemplars.

Moreover, although the present study provides new insights into the attribution of human social constructs to robots, the method of investigation remains purely perceptual and does not make it possible to draw conclusions about the strength of the “made-in effect” in real HRI. While there is evidence about an in/out-group bias in HRI (Eyssel and Kuchenbrandt, 2012), similar effects could occur for different out-groups depending on the positive vs. negative valence of the stereotypes associated with them. Further research will need to address this point before it is possible to draw a conclusion. HRI studies of this type are essential if we are to understand why people might adopt unethical behaviors with regard to robots—not necessarily in order to protect robots but in order to safeguard human morality. Could modern society accept that humans harm robots because they have a certain shape or country of manufacture? Might this have consequences for our own human social constructs by validating stereotypes? Or might it even create new stereotypes because certain countries provide robots for behaviors that are prohibited with other humans?

In line with the literature, one result of the present study is the consistency of the finding of a greater attribution of warmth, competence, human uniqueness, and human nature traits to the two robots having a face. This effect is a standard finding in the study of humanoid robotics (Duffy, 2003; Jadeja, 2008). The presence of certain features (e.g., eyes, mouth, nose, skin, size homogeneity) greatly influences the perception of anthropomorphic traits in robot heads. Irrespective of the country of origin, these facial features seem to be omnipresent in the evaluation of robots. This factor should be controlled for in further studies in order to test whether different anthropomorphic faces, constructed using different variables, can modulate the attribution of human traits and social constructs to robots. It is possible that some facial features elicit social and national stereotypes to a greater extent and consequently determine the quality of HRI.

Finally, based on the present findings, the psychological outcomes of granting citizenship to robots should be further investigated. One may wonder how discordant information about a robot's country of manufacture and its citizenship might impact individuals' perception of the robot. The recent example of the robot Sophia, which was granted Saudi-Arabian citizenship even though being designed in Hong-Kong, is intriguing. In the light of the social psychology literature, one could argue that the perception of Sophia might have changed before and after being granted citizenship but that its “made in Hong-Kong” label might have moderated this effect.

## Conclusion

The present study argues that social categories represent one of the most important determinants of how humans evaluate and perceive robots. Stereotypical human contents can be projected onto robots. More than an in-group/out-group phenomenon, differences in nationality can impact the conceptual distance between artificial entities and humans. Therefore, it seems that, rather than being influenced by anthropomorphism in general, individuals tend to assimilate these new artificial agents to their own species, thus resulting in similar social cognition biases such as implicit associations (i.e., information acquired and used unconsciously, and affecting thoughts and behaviors). Furthermore, the representation of countries may vary depending on geographical location, and we therefore assumed that the cultural perception of the “made-in effect” in the case of robots might fluctuate depending on the observers' culture of origin. Despite this, warmth and competence are reliable universal dimensions of social judgment. If the attribution of a nationality can modify the attribution of human traits to robots, it is necessary to raise the question of the attribution of citizenship or the presentation of the place of assembly of robots. Particular combinations of warmth/competence have distinct emotional and behavioral consequences (Fiske et al., 2007) that should be taken into account in the case of HRI. This is a particularly relevant issue in terms of how we will interact with robots and it could explain the motivation to reduce the distance between these new artificial agents and us. This issue is not futuristic considering that the robot Sophia has been granted Saudi Arabian citizenship. In addition to the related political and ethical questions, social scientists also have to question the impact of the granting of nationality on the perception of robots.

## Ethics Statement

This study was approved by the Clermont-Ferrand Sud-Est 6 Statutory Ethics Committee (Comité de Protection des Personnes (CPP) Sud-Est 6, France) and was carried out in accordance with the provisions of the World Medical Association Declaration of Helsinki.

## Author Contributions

All authors listed have made a substantial, direct and intellectual contribution to the work, and approved it for publication.

### Conflict of Interest Statement

The authors declare that the research was conducted in the absence of any commercial or financial relationships that could be construed as a potential conflict of interest.

## Footnotes

^1^All participants were French students, our results might not generalize to other students (English or Japanese for instance). This remains indeed an empirical question.

## Supplementary Material

Supplementary Presentation 1 Pretest countries selection according to warmth and competence attributions.

Supplementary Table 1 National stereotypes and robots' perception: the “made in” effect open data.
