# Supplementary material for: National Stereotypes and Robots' Perception: The “Made in” Effect
Source: Front Robot AI. 2019 Apr 9;6:21. doi: 10.3389/frobt.2019.00021 (PMC7805950; doi:10.3389/frobt.2019.00021)
Supplement: Supplementary Presentation 1 — Pretest countries selection according to warmth and competence attributions. [file Presentation_1.pdf]

Supplementary materials

1. Pretest

In the person perception domain, Rosenberg, Nelson and Vivekananthan [1] were the first to provide evidence of the presence of two dimensions that organize how we perceive others in terms of personality traits. To name these two dimensions Fiske, Cuddy, Glick and Xu [2] used the labels warmth and competence. These two dimensions are used as the basis of stereotypes' intergroup functions. To select the foreign countries taking count of these psychological determinants of our social perception we designed a pre-test. Fiske et al. [3], proposed that these two dimension could be combined to give an account of intergroup stereotypes with a low/high warmth and a low/high competence (e.g. high competence with low warmth combination).

2. Method

Qualtrics Survey online software was used to design and distribute the questionnaire. Countries have been chosen according to the 20 first country with the highest PIB [4]. We adapted the competence and warmth trait questionnaire from Kervyn, Yzerbyt, Demoulin and Judd [5] to a 6 point likert scale. “Competence” was computed from *ambitious*, *competent*, *intelligent*, *assured*, *efficient*, *intent* traits while “Warmth” was computed from *moral*, *sociable*, *warm*, *honest*, *likeability*, *trustworthy* traits. Each trait was presented in a random order and participant had to evaluate each 20 countries (Germany, Japan, Switzerland, Russia, United-Kingdom, Canada, China, United States, Australia, Netherlands, South Korea, Saudi Arabia, France, Italy, India, Brazil, Turkey, Spain, Indonesia, Mexico) on each trait. Participants judged all traits for all countries.

3. Results

Participants were 20 women and 17 men (Mean age = 25.5) recruited online in France. In order to take account of the in-group bias, we used the scores for France as the central point for the graphic.

We used the mixed matrix of the two primary dimensions of competence and warmth proposed by Fiske, Cuddy, Glick, & Xu [2] with high/low warmth and high/low competence to determine the more representative country for each matrix conceptual position (Figure 1).

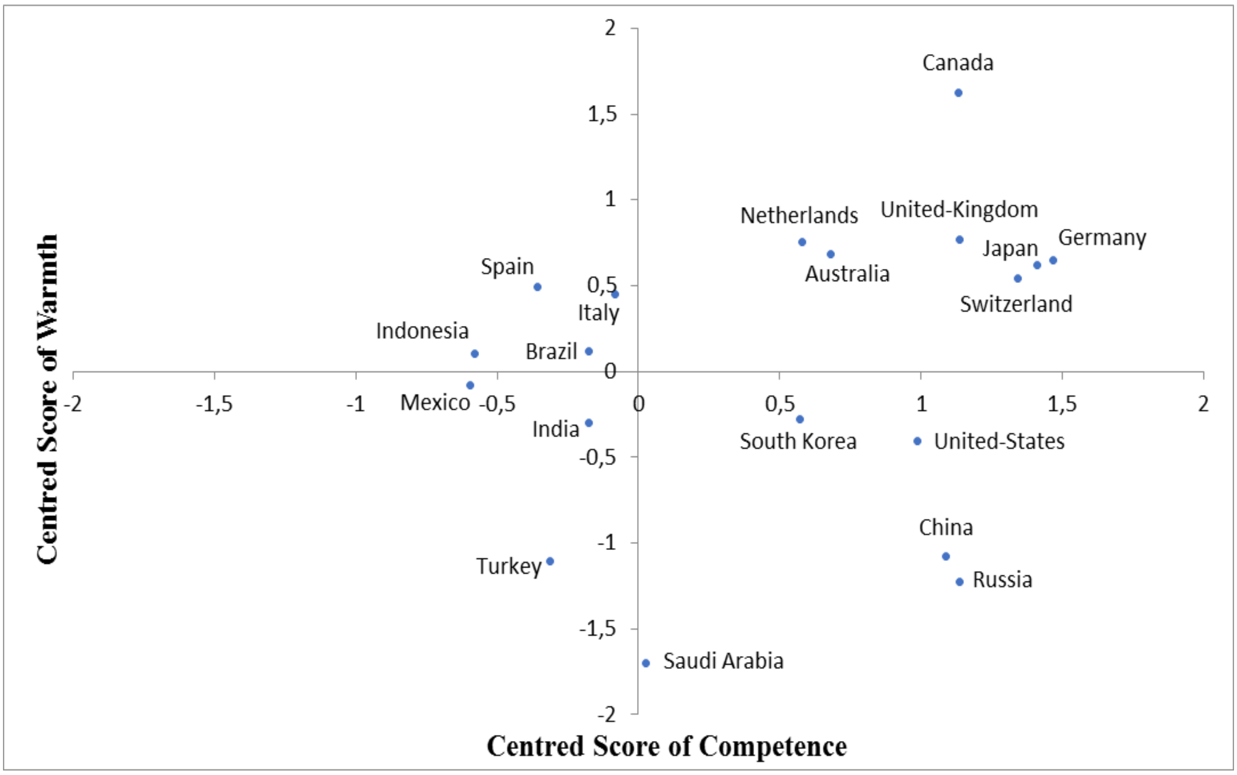

Fig. 1. Countries on Warmth (x) and Competence (y) dimension with France (participant's country) as intercept.

4. Supplementary references.

1. Rosenberg S, Carnot N, Vivekananthan PS. A multidimensional approach to the structure of personality impressions. J Pers Soc Psychol. 1968; doi:10.1037/h0026086
2. Fiske ST, Cuddy AJC, Glick P, Xu J. A model of (often mixed) stereotype content: Competence and warmth respectively follow from perceived status and competition. Social Cognition: Selected Works of Susan Fiske. 2018. doi:10.4324/9781315187280
3. Fiske ST, Cuddy AJC, Glick P. Universal dimensions of social cognition: warmth and competence. Trends Cogn Sci. 2007;11: 77–83. doi:10.1016/j.tics.2006.11.005
4. The World Bank. GDP Growth (Annual %). In: World Bank Data. 2014. doi:10.1017/CBO9781107415324.004
5. Kervyn N, Yzerbyt V, Judd CM. Compensation between warmth and competence: Antecedents and consequences of a negative relation between the two fundamental dimensions of social perception. Eur Rev Soc Psychol. 2010; doi:10.1080/13546805.2010.517997
